# Risk factors for infection with carbapenem-resistant Klebsiella pneumoniae: a case-case-control study

**Published:** 2014-06-30

**Authors:** Viviana Gómez Rueda, John Jairo Zuleta Tobón

**Affiliations:** Clinical Epidemiology Group, Universidad de Antioquia, Medellín, Antioquia, Colombia; GRAEPIC, Research Unit, Hospital Pablo Tobón Uribe, Medellín, Antioquia, Colombia

**Keywords:** Klebsiella pneumoniae, carbapenems, case-control studies, risk factors, quinolones

## Abstract

**Objetive:**

To evaluate the association between quinolone exposure and the emergence of carbapenem-resistant *Klebsiella pneumoniae* (CRKP) and to estimate CRKP-specific mortality.

**Methods:**

Case-case-control study implemented in a tertiary care institution. Three groups of patients were analyzed: 61 consecutive cases of infection with CRKP (Group I); 61 randomly chosen cases of patients infected with carbapenem-sensitive *Klebsiella pneumoniae* (CSKP; Group II); and 122 randomly chosen controls without CRKP or CSKP infection. Matching was based on the length of stay in intensive care unit and the date of bacterial isolation. An active search was performed for patients with CRKP and CSKP infection, and prospective cases were included in the study. We compared the results for Groups I and II against those for the controls by using two conditional logistic regression analyses that included infection as the dependent variable and controlled for time at risk and comorbidities.

**Results:**

Exposure to quinolones was not associated with CRKP infection: no association was found in the analysis of CRKP with the controls (OR= 1.7; 95% CI: 0.2-6.5) or in the analysis of CSKP against the controls (OR= 0.6; 95% CI: 0.2-1.6). Use of carbapenems (OR= 3.3; 95% CI: 1.2-9.3) and colonization with CRKP (OR= 3.3; 95% IC: 1.2-9.3) were specific risk factors for infection with CRKP. Mortality associated with CRKP was 61.3%.

**Conclusion:**

No association was found between exposure to quinolones and infection with CRKP; however, colonization by CRKP and use of carbapenems are risk factors for infection with CRKP.

## Abstract (es)

**Objetivo:**

Evaluar la asociación entre la exposición a quinolonas y la aparición de infección por *Klebsiella pneumoniae *resistente a Carbapenémicos (CRKP) y estimar la mortalidad específica por CRKP.

**Métodos:**

Estudio caso-caso-control realizado en una institución de tercer nivel de atención. Se adelantó búsqueda activa y prospectiva de los casos. Se analizaron tres grupos: 61 casos consecutivos de infección por CRKP, 61 casos elegidos al azar de los pacientes con infección por *Klebsiella pneumoniae* sensible a Carbapenémicos (CSKP) y 122 controles sin infección por CRKP ni por CSKP, elegidos al azar. Se realizó emparejamiento por estancia en unidad de cuidados intensivos y fecha del aislamiento bacteriano. Los datos se extractaron de la historia clínica electrónica. Se comparó los casos CRKP y los casos CSKP contra los controles mediante dos análisis de regresión logística condicional, con la infección como variable dependiente y controlando por el tiempo en riesgo y la comorbilidad.

**Resultados:**

La exposición a quinolonas no se asoció a infección con CRKP: no se encontró asociación en el análisis de CRKP contra controles (OR 1.7 IC 95%: 0.2-6.5) ni en el análisis de CSKP contra controles (OR 0.6 IC 95%: 0.2-1.6). El uso de carbapenémicos (OR 3.3 IC 95%: 1.2-9.3) y la colonización por KPRC (OR 16.2 IC95%: IC95%:3.3-79.1) fueron factores de riesgo específicos para infección por CRKP. La mortalidad específica asociada a CRKP fue de 61.3%.

**Conclusión:**

No se encontró asociación entre la exposición a quinolonas y la infección por KPRC, pero la colonización por CRKP y el uso de carbapenémicos son factores de riesgo asociados a la infección por KPRC.

## Introduction

Ever since the first isolation of carbapenem-resistant *Klebsiella pneumoniae* (CRKP) in the United States during the 1990s, its incidence has been on the rise worldwide with increased mortality, morbidity, length of hospital stay, and associated costs 1. The emergence of resistance to carbapenems is a challenge due to the limited number of antimicrobials available to treat this type of infection. In Colombia, CRKP was first detected in 2005 2 and Colombia is currently listed as a country with endemic and epidemic conditions within the global distribution of CRKP 3. 

Exposure to antibiotics is a risk factor for healthcare-associated infections (HAIs) caused by multi-resistant bacteria through the selection of endogenous flora and simultaneous exposure to antibiotics in hospital environments and in patients. Recently published studies have documented that exposure to quinolones is a risk factor for infection with CRKP 4 ^,^ 5 ; however, other studies have not found this association or have suggested a protective effect of quinolone use 1 ^,^ 6. Several authors have proposed case-case-control studies to determine the risk factors for infection with resistant bacteria 7 ^-^ 9, which is a methodology that has been infrequently used in studies on CRKP infections 4 ^,^ 10 ^,^ 11. We conducted a case-case-control study to determine whether exposure to quinolones is a risk factor for CRKP infection in hospitalized patients and to estimate the attributed mortality associated with CRKP infections.

## Materials and Methods

### Study design

This study was a case-case-control analysis matched by the length of stay in intensive care unit (ICU) and the date of bacterial isolation. Patients who were infected with CRKP (Group I) were compared to a sample of patients who were hospitalized without infection (controls) to determine the risk factors for CRKP and carbapenem-sensitive *Klebsiella pneumonia* (CSKP) infections. Patients infected with CSKP (Group II) were compared to the same sample of uninfected hospitalized patients (controls) to determine the risk factors for CSKP infection. These patients represent models of risk that enable estimating the specific risks for CRKP infection 8.

### Study population and characteristics

The study population included patients hospitalized from January 2008 through January 2011 at Hospital Pablo Tobón Uribe (HPTU), a tertiary teaching hospital in Medellín Colombia, with 350 beds and 11,500 annual discharges. The total number of patients infected with CRKP was included, beginning with the index case, which was captured in the hospital's database of the infection prevention committee. The CSKP-infected patients were randomly selected from a list of patients that is kept in the WHONET program (World Health Organization, Geneva, Switzerland, version 2.0, 2010) by the microbiology laboratory. Antimicrobial susceptibilities were determined between January 2008 and October 2009 by the disc diffusion method and between November 2009 and January 2011 by the VITEK ^®^ 2 Compact System (bioMérieux, Lyon, France) in keeping with the Clinical and Laboratory Standards Institute (CLSI). Sixty two clinical isolates of *K. pneumoniae* exhibited resistance to imipenem, meropenem and/or ertapenem and all clinical isolates were confirmed by the modified Hodge test. Clinical cultures were processed according to routine bacteriological procedures, and *K. pneumoniae* strains were identified based on the CLSI recommendations. When a strain was found to be resistant to ertapenem or a discrepancy was observed among the susceptibility to different carbapenems, a modified Hodge test was performed to confirm the finding. Infection diagnosis was made according to HAI criteria from the Centers for Disease Control and Prevention (CDC) in the United States. For Groups I and II, the infection was diagnosed after 48 h of hospitalization. A total of two control patients were included per patient in Group I for a total of 122 controls. The control patients were hospitalized for more than 48 h, did not have CRKP or CSKP infection, and were randomly selected from a list of hospital discharges during the study period. The controls were the same for both groups. The clinical and demographic data were obtained from the Institution´s electronic medical records. Details on the kind of carbapenemase are not available because molecular epidemiology investigations using pulsed-field gel electrophoresis patterns or PCR for resistance genes or sequencing of PCR products were not performed in our study.

We analyzed the demographic characteristics (age and sex), hospitalization characteristics (frequency of ICU stays, length of ICU stay in days, number of hospitalizations within the previous six months, and referrals from other institutions), features related to devices and interventions (use of central venous catheters, parenteral nutrition, mechanical ventilation, indwelling urinary catheters, gastrostomy and tracheostomy during hospitalization, and a history of surgery within the previous month), colonization by CRKP and concomitant bacterial infections, immunosuppression (transplant history, use of systemic steroids, use of immunomodulators, Like cyclosporine, tacrolimus, azathioprine, mycophenolate, cyclophosphamide, methotrexate, infliximab, etanercept, sirolimus, chloroquine, chemotherapy, or radiotherapy), and the class of antibiotics administered. Oral or parenteral antibiotics were administered as at least one dose within 14 days prior to bacterial isolation for patients with CRKP or CSKP infection and within 14 days prior to discharge for the controls. The CRKP colonization was considered when a patient had positive CRKP surveillance cultures without clinical signs of infection. The time at risk was calculated for the CRKP and CSKP cases as the number of days between the date of admission and the date of bacterial isolation and as the number of days from the date of admission to the date of discharge from the institution for the controls. Co-morbidities were evaluated by using the Charlson Index 12. Risk of infection according to the other factors was considered from the date of hospital admission until the diagnosis of infection for Groups I and II and until discharge for the controls.

###  Analysis

The categorical variables were expressed in absolute and relative numbers. The assumption of normality for the continuous quantitative variables was verified with the Kolmogorov-Smirnov test, and the values were presented as the mean, standard deviation or median (Me), and percentiles. We calculated the odds ratios (ORs) and the respective confidence intervals (CIs) for the dichotomous categorical variables with CRKP or CSKP infection as the dependent variable. *P* values were calculated with Pearson's chi-squared test for the qualitative variables with expected values greater than 5. In case of frequencies of less than 5, the *p* values were calculated by using a chi-squared test with a continuity correction. For the continuous variables with normal distribution, Student's *t*-test was performed, and a Mann-Whitney U test was used when this assumption was not met.

We performed two conditional logistic regression analyses using the EGRET program: one analysis with CRKP infection as the dependent variable and the other analysis with CSKP infection as the dependent variable. The main independent variable was exposure to quinolones. Comorbidities and the time at risk are confounding factors because they are associated with an increased risk of infection and exposure to antibiotics; therefore, these variables were included in all of the adjustment models 7. A total of four other variables were included in the multivariate model of the first analysis, which were selected from a list of potential risk factors proposed before the analysis and which had a *p* value of less than 0.1 in the bivariate analysis. The variables were evaluated to exclude multi-collinearity and interaction. The main independent variable, confounding variables, and independent variables that retained a value of *p *<0.05 were included in the model. The second model included the same variables from the first model to enable comparison between the two analyses.

The attributed mortality was calculated by subtracting the mortality of patients infected with CRKP from the mortality of patients infected with CSKP 13. Additionally, two infectious disease specialists at the hospital independently evaluated the cases to qualify the relationship between mortality and CRKP infection according to a routine epidemiological surveillance process. When there was disagreement, a third infectious disease specialist reviewed the history and a decision was made by consensus. The evolution of the underlying disease, presence of co-morbidities, type of treatment, and potential causal relationship between the CRKP infection and the direct cause of death were taken into account.

This study was approved by the Hospital´s Research and Ethics Committee. This study complies with Resolution 8430 of 1993 from the Colombian Ministry of Social Protection.

##  Results

A total of 76 patients were found to be infected with CRKP; however, 14 patients were diagnosed within 48 h of hospitalization, and the respective controls were not found for one patient. Consequently, the study included 61 cases of infection. The diagnoses of these patients were the following: 15 (24.59%) cases of urinary tract infection, 13 (21.31%) cases of pneumonia, 12 (19.67%) cases of bacteremia, 10 (16.39%) cases of peritonitis, 6 (9.83%) cases of soft tissue infection, 2 (3.27%) cases of chronic osteomyelitis, 1 (1.63%) case of cerebritis, and 1 (1.63%) case of septic arthritis.

The minimum inhibitory concentration distribution for 18 patients evaluated by the VITEK^®^ 2 Compact System from November 2009 are presented in supplementary Table 1.

**Table 1.**
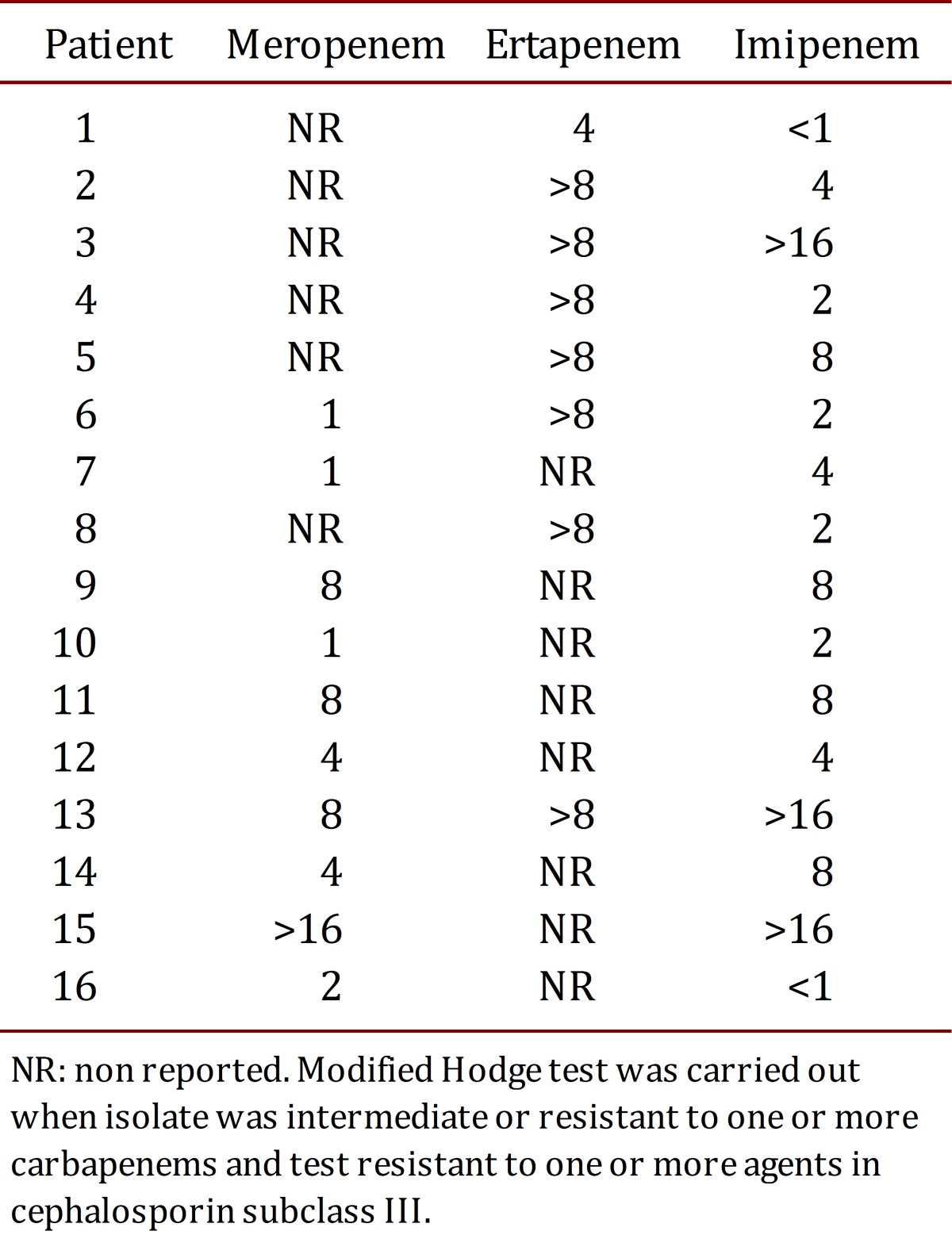
Distribution of the minimum inhibitory concentration for 16 CRKP isolates

### Analysis of the CRKP-infected patients vs. controls

The main characteristics of the study population are shown in Table 2. In the univariate analysis, a difference was noted in the time at risk between the groups, which was greater in patients infected with CRKP. The Charlson Index was similar in both groups. Because of the matching, the frequencies of ICU stay were similar; however, the number of days in the ICU was significantly higher in patients infected with CRKP during the previous 14 days and the entire stay in this unit. A significant difference was found in the history of previous hospitalizations within the previous six months. Cases with previous hospitalization were more exposed to invasive devices, used immunomodulators, and had a history of transplantation, chemotherapy, or antibiotic use. Devices and interventions indicated increased risk of infection, but only the use of enteral or parenteral nutrition and central venous catheter use were statistically significant. No differences were found among the groups according to the history of transplantation, but a difference was noted in the use of immunomodulatory agents. Colonization with CRKP increased the risk of CRKP infection by 17-fold. In these cases, use of antibiotics was more frequent and the number of antibiotics used was greater; nevertheless, the only differences were found in the use of piperacillin-tazobactam, carbapenems, and linezolid. Use of quinolones was proportionally greater in patients with CRKP infections but not statistically significant.

**Table 2.**
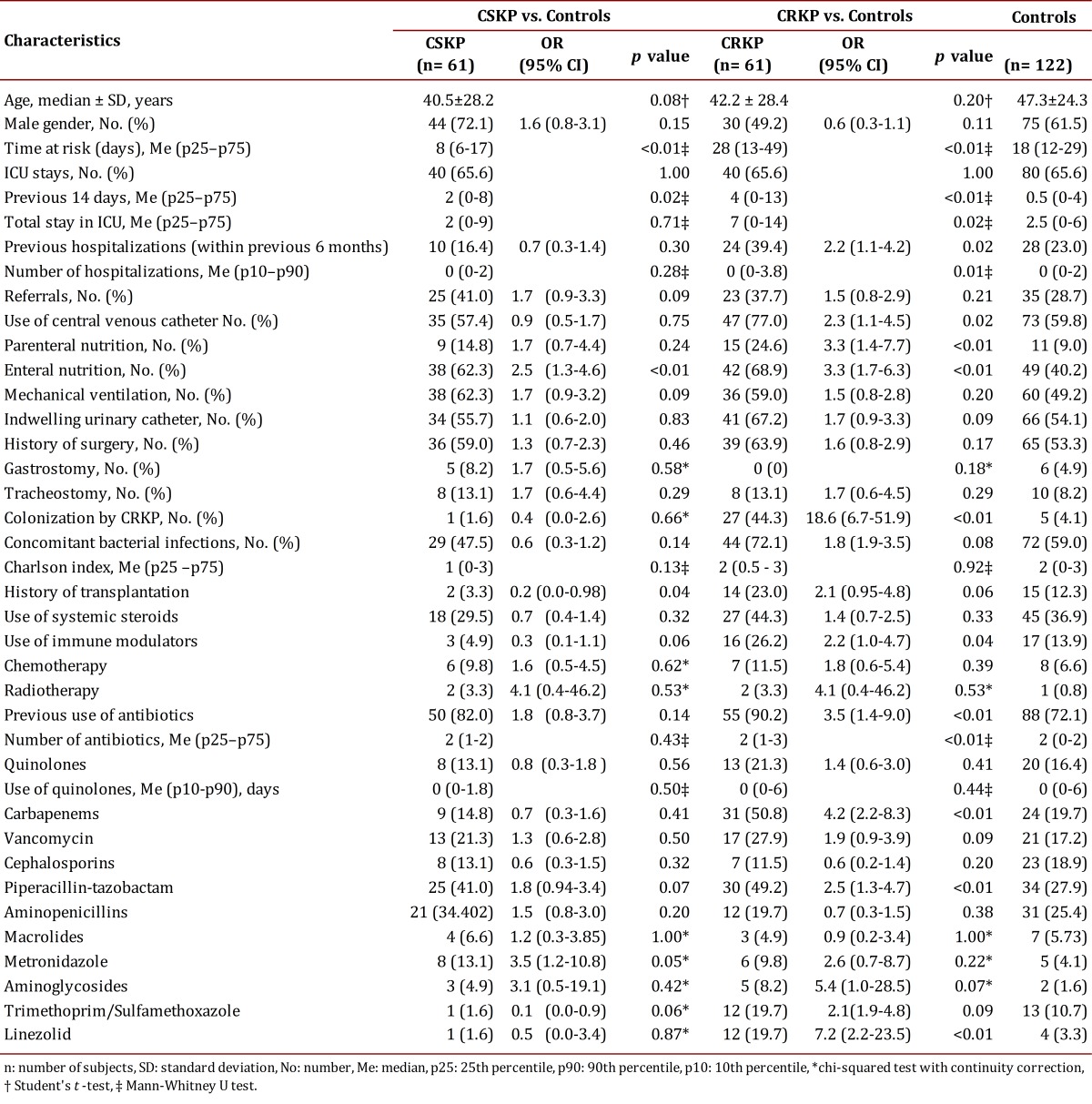
Factors associated with infection with *Klebsiella pneumoniae *(KP) and carbapenem-sensitive *Klebsiella pneumoniae* (CSKP)

###  Analysis of the CSKP-infected patients vs. controls

In the univariate analysis, the time at risk was significantly greater in the controls compared to patients infected with CSKP; however, the length of ICU stay was greater in patients infected with CSKP. The number of previous hospitalizations and the Charlson Comorbidity Index was proportionally higher in controls. There were a higher number of CSKP-infected patients exposed to enteral nutrition, parenteral nutrition, and mechanical ventilation; however, only exposure to enteral nutrition was statistically significant. The frequency of central venous catheter use was greater in the controls. Use of immunosuppressing agents and a history of transplantation were more frequent in the controls, but no statistically significant differences were identified between the groups. Use of chemotherapy and radiation therapy was more frequent in patients infected with CSKP. Use of antibiotics was similar in both groups. The results are reported in Table 2.

### Comparison of the two models

Table 3 shows the results adjusted for the selected variables. Use of piperacillin-tazobactam and enteral nutrition was associated with infection in the two models, whereas use of carbapenems and colonization by CRKP was associated with infection in the model that compared CRKP with the controls. Quinolone exposure was not found to be associated with infection in the two models.

**Table 3.**
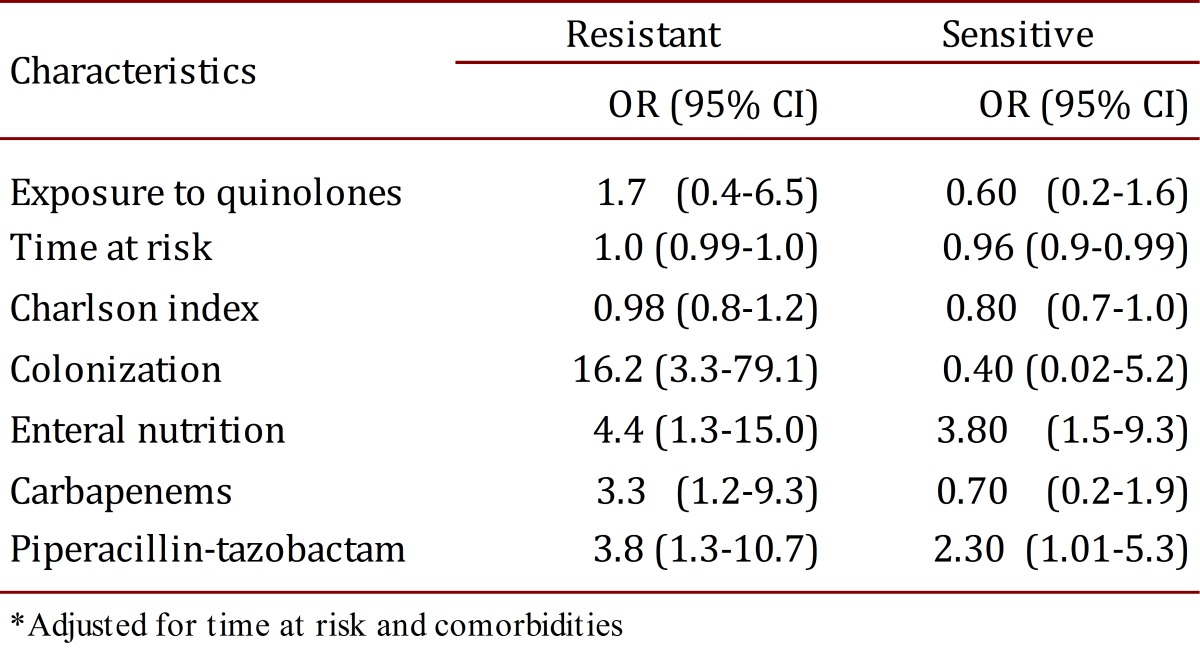
Conditional logistic regression of the risk factors for infection with *Klebsiella pneumonia*

### Mortality 

Overall, 31 (50.8%) patients infected with CRKP and 20 (32.7%) patients infected with CSKP died. The crude mortality rate of the controls was 20.4% (25). Mortality that was attributable to CRKP infection was 18.1% (11). In the individual case analysis, which was conducted by infectious disease specialists, 19 of the 31 deaths (61.3%) were considered attributable to infection with CRKP.

## Discussion

This study found no association between exposure to quinolones and infection by CRKP; however, there was an association between exposure to carbapenems and CRKP infection. In the literature, contradictory findings point to the relationship between the use of these antibiotics and resistance to carbapenems.

Three studies comparing patients infected with CRKP to patients infected with CSKP found association between quinolones and the risk of infection 2 ^,^ 5 ^,^ 7, whereas three other studies, including one study of patients with bacteremia, did not find this association 6 ^,^ 14 ^,^ 15. In these studies, the inclusion of patients infected with susceptible organisms as controls generated a selection bias because a minority of the patients infected with resistant bacteria came from a cohort of patients infected with antibiotic-sensitive bacteria, and this study overestimated the effect of antibiotics because their use inhibits the growth of susceptible microorganisms 7. An additional study comparing patients infected with CRKP to hospitalized patients found that prior use of quinolones was a protective factor 11. 

Several studies have demonstrated the superiority of the case-case-control methodology over the case-control methodology in the study of the risk factors for resistant organisms while recognizing their limitations 16 ^,^ 17. This methodology has been recommended by different authors 9 ^,^ 18 ^,^ 19 and has been employed to study risk factors for other microorganisms. We found three studies on risk factors for CRKP infection that using this methodology 4 ^,^ 10 ^,^ 20. The first study was conducted in Israel and found that the only risk factor for CRKP infection was prior use of antibiotics, especially quinolones and carbapenems^4^. The second study was conducted in Puerto Rico and did not find any risk factors for CRKP infection after performing adjusted analyses and comparing the two models 20. The third study was conducted in Greece and found that increased duration of quinolone use was associated with infection in the model that evaluated CSKP and the model that evaluated CRKP; therefore, quinolone use is not a unique risk factor for CRKP 10. Our study and these studies used the case-case-control methodology; however, there are differences in the adjustments for the time at risk, comorbidities, and the definition of variables, which may explain the divergent findings.

The definition of time of exposure to antibiotics was different among the studies. Exposure time in our study was 14 days before a diagnosis in cases of infection or on discharge for the controls. In other studies, the times were 30 days 20, six months 10, or throughout hospitalization regardless of the duration 4. These differences can account for the divergent findings. The studies that evaluated the risk factors for multi-resistance indicated significant variability in the windows of exposure time; the majority of the studies defined this time as a categorized variable with different thresholds of the minimum duration of antibiotic use. Other studies analyzed time of exposure as a continuous variable or as a categorized variable. Therefore, no agreement has been reached on the appropriate method to evaluate time of exposure.

To identify the risk factors associated with multi-resistant bacterial infections, the authors of the previous studies recommend adjusting for the presence of comorbidities. In our study, we included the absolute value of co-morbidity measurements into the regression models. In one of the published case-case-control studies, diseases were included as independent variables in the statistical models 13. In another study, the Charlson Co-morbidity Index was used to categorize the degree of comorbidity as high or low 4. In the third study, the co-morbidity value was integrated into a prognostic index, which was added to other variables 10. These adjustments represent different methods of analyzing the data; yet, these methods are all valid alternatives and would not account for the differences in the results.

In this study, use of carbapenems was a specific risk factor for CRKP, which is consistent with the results of two out of three case-case-control studies 4 ^,^ 10. The evolution and spread of antibiotic resistance are related to the antibiotic pressure exerted on the microbial environment and exposure to different concentrations of antibiotics. Antibiotic pressure, together with the high rate of spontaneous genetic variation and bacterial survival, selects for resistant strains in internal and external environments. When more than one antibiotic is present in the bacterial environment, pressure from these antibiotics results in the selection of bacteria that use multiple or polyvalent resistance mechanisms. Within this context, bacteria optimize one resistance mechanism to survive in variable environmental conditions or increase mutational events during situations of bacterial stress 21.

This study demonstrated that enteral nutrition is significantly associated with KP infection. This finding may be explained by the weakening of the mucous membranes of the digestive system, a modification of commensal intestinal flora, and bacterial translocation from the gastrointestinal tract into the general circulation, which is produced by this type of nutrition 22. Similarly, colonization by CRKP is a risk factor for infection with CRKP, which has been underreported in investigations on this microorganism. A descriptive study on the use of active epidemiological surveillance cultures for CRKP stated the need for additional studies to detect asymptomatic colonization and diagnose infection after colonization 23. Two recent case-control studies identified the risk factors for colonization by CRKP 4 and risk factors for infection with CRKP in patients who were previously colonized by this microorganism 24. Other studies have described the role of colonization by other microorganisms 25. 

The presence of antibiotic resistance in bacterial strains increases mortality. In our study, the attributed mortality of CRKP infection was 18.1%; however, when the clinical judgments of the infectious disease specialists were included in the analysis, this mortality increased to 61%. These findings are similar to those found in other studies, which reported a two- to three-fold increase in the mortality of CRKP infection, with crude mortality rates between 48 and 71.9% 6 ^,^ 13. 

An important strength of this study is the control of two variables that have been associated with confounding bias in case-case-control studies: the time at risk and comorbidities 7. In addition, we performed a pairing for ICU stay, which helped to control for other potential confounders, such as complex and invasive procedures or the exposure to flora that were selected by antibiotic pressure. Moreover, the case-case-control model allows us to obtain the specific risk factors for the resistant microbes. Similarly, the study has several limitations. The CRKP infection was a recent outbreak at our Institution; therefore, a fixed sample of cases was obtained, which limits the power and precision of the study and identification of other risk factors associated with a low frequency of exposure. Moreover, three related clones and 14 unrelated clones were identified at this institution, including KPC-2 and KPC-3 strain types; nonetheless, molecular identification was not obtained for all of the strains. These molecular mechanisms could be associated with different risk factors. Another limitation is the difficulty in verifying the role of patient-to-patient transmission. A well-documented potential bias in this type of study is that patients with the susceptible organism are not included in the control group for patients with resistant organisms, and resistant infections are not included in the control group for patients infected with antibiotic-sensitive bacteria, which contradicts the basic principle of study populations in case-control study designs. However, it has been estimated that these excluded patients represent a fraction of less than 1% of the population from which the control group is selected; therefore, this bias minimally affects the estimation of the results 8. Additional cohort studies are needed with larger sample sizes and longer follow-ups to clarify the discrepancies of these studies.

## Conclusion

 To prevent and control outbreaks caused by CRKP, surveillance cultures must be continued and antibiotics must be administered reasonably. Similarly, timely and appropriate antibiotic treatment should be initiated for CRKP-infected patients who have been previously colonized by CRKP.
